# Engineered human organ-specific urethra as a functional substitute

**DOI:** 10.1038/s41598-022-25311-1

**Published:** 2022-12-09

**Authors:** Christophe Caneparo, Stéphane Chabaud, Julie Fradette, Stéphane Bolduc

**Affiliations:** 1 LOEX/Regenerative Medicine Division, CHU de Québec-Université Laval Research Center, Hôpital Enfant-Jésus, 1401, 18E Rue, Quebec, QC G1J 1Z4 Canada; 2 Department of Surgery, Faculty of Medicine, Université Laval, Quebec, QC Canada

**Keywords:** Biological techniques, Biological models, Immunological techniques, Microscopy, Cells, Urinary tract, Bladder, Paediatric urology, Ureter, Urethra

## Abstract

Urologic patients may be affected by pathologies requiring surgical reconstruction to re-establish a normal function. The lack of autologous tissues to reconstruct the urethra led clinicians toward new solutions, such as tissue engineering. Tridimensional tissues were produced and characterized from a clinical perspective. The balance was optimized between increasing the mechanical resistance of urethral-engineered tissue and preserving the urothelium’s barrier function, essential to avoid urine extravasation and subsequent inflammation and fibrosis. The substitutes produced using a mix of vesical (VF) and dermal fibroblasts (DF) in either 90%:10% or 80%:20% showed mechanical resistance values comparable to human native bladder tissue while maintaining functionality. The presence of mature urothelium markers such as uroplakins and tight junctions were documented. All substitutes showed similar histological features except for the noticeable decrease in polysaccharide globules for the substitutes made with a higher proportion of DF. The degree of maturation evaluated with electron microscopy was positively correlated with the increased concentration of VF in the stroma. Substitutes produced with VF and at least 10% of DF showed sufficient mechanical resistance to withstand surgeon manipulation and high functionality, which may improve long-term patients’ quality of life, representing a great future alternative to current treatments.

## Introduction

Urologic patients may be affected by pathologies requiring surgical reconstruction to re-establish a normal function. Among them, hypospadias and urethral strictures are the most common penile pathologies. Hypospadias is a mislocalization of the urinary meatus along the ventral aspect of the penis. This anomaly affects one boy over 250, and its prevalence is increasing, mainly due to endocrine disruptors in our environment^1–4^. Proximal hypospadias, i.e. meatus near the scrotum, is the most severe form, whereas distal hypospadias, i.e. meatus near the normal position (closed to the glans), is the less severe form. Urethral stricture is a narrowing of the urethra in its anterior part, which can result from various causes, including trauma, infectious diseases, and surgeries. The stricture can be partial or total; in this case, it is an emergency condition. Unfortunately, few tissues can be used to repair or replace the urethra. Skin grafts, including genital and extragenital skin flaps, *tunica vaginalis* around the testicles, and lingual or buccal mucosa, have been tested^5–13^. Oral mucosa remains today the gold standard^14^.

Nevertheless, this approach leads to potential short- and long-term complications, ranging from re-stenosis, numbness, fistulae, submucosal scars, dry mouth, difficulty opening the mouth due to contracture, neuro-sensory defects, lesions, discomfort, pain and risk of infection^12,15,16^. It should also be noted that the amount of tissue that can be harvested is limited, which is problematic for severe and recurrent cases^16–21^. These limitations lead clinicians to develop new approaches.

Tissue engineering (TE) is a promising regenerative medicine strategy to overcome these challenges. The goal is to provide functional in vitro engineered substitutes to restore structure and function to altered tissues. For example, different biomaterials have been explored for urethral reconstruction^22–27^. Unfortunately, despite numerous associated potential advantages, synthetic or biological scaffolds commonly lead to long-term complications with adverse clinical outcomes^7,28–33^.

Recently, urologic TE was approached from a different angle. Instead of using a preexisting scaffold on which cells are seeded, the self-assembly technique uses mesenchymal cells, such as fibroblasts, to secrete and assemble endogenous extracellular matrix (ECM), allowing the production of tissues devoid of exogenous biomaterial^34^. This technique produced urethral substitutes using dermal fibroblasts (DF). These substitutes have higher mechanical characteristics than native porcine urethra^35^. In addition, mechanical stimulation can improve the epithelial differentiation bringing functionality (waterproofness) to the substitutes^36^, which can also be endothelialized if needed^37^. This engineered tissue is integrated into host tissue after grafting into nude mice's backs and can benefit from prevascularization^37^. However, all these substitutes made with DF showed the presence of epidermal markers in the urologic epithelium, the urothelium. As previously demonstrated with other organs^38^, using “organ-specific” cells, i.e. vesical fibroblasts (VF) to produce urologic substitutes, allowed expression of urothelial markers more closely resembling the native tissue compared to substitutes produced with DF^39^.

However, the mechanical properties of tissues produced with VF were too weak to be used for clinical application^39^. It was, therefore, necessary to find a solution to optimize the balance between adequate mechanical resistance of the engineered tissues and preservation of the epithelium’s barrier function, essential to avoid internal urine leakage (extravasation) and subsequent inflammation and fibrosis. To reach this goal, we tested the hypothesis that a substitute featuring optimal structural and functional properties could be engineered by mixing a small proportion of DF (to improve mechanical resistance) with VF (supporting a better epithelium) during in vitro reconstruction.

The objectives were to obtain at least the mechanical resistance found in the native human urethral mucosa with the same degree of epithelial maturation and, therefore, the corresponding functionality. Unfortunately, no data from uniaxial tensile testing (for human native urethra) has been found in the literature, despite that biochemical behaviour of mal human urethral tissues has been characterized^40^. However, human bladder mechanical properties have been characterized by a maximal strength and elastic modulus, respectively of 0.27 N and 0.25 MPa^41^. Therefore, these represent the reference values to estimate the mechanical resistance necessary to obtain a manipulable substitute rather than the value one wants to compare the urethra with the bladder.

## Results

### The hybrid assembly technique improved the mechanical properties of the flat VF urethral tissues

**Figure 1:**
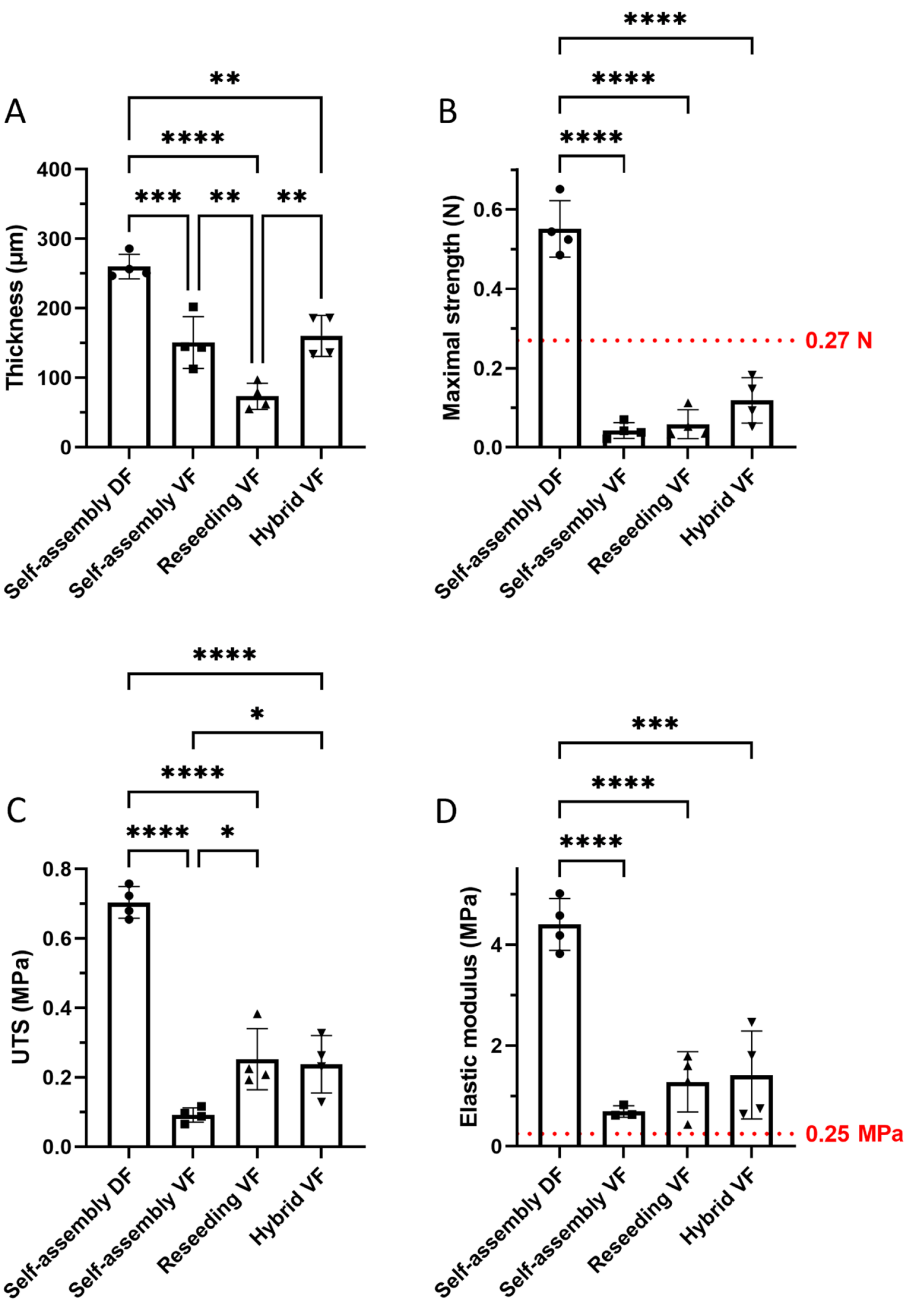
Mechanical assessment of the substitutes according to the engineering method. Each symbol represents the mean of measurements obtained from one urethral substitute. The bars represent the mean +/− the standard deviation of the means of reconstructed urethra substitutes. Self-assembly DF is for the condition where the stroma of the urethra substitutes was reconstructed using only dermal fibroblasts (DF) and the standard self-assembly protocol (i.e. three stromal sheets stacked together). Self-assembly VF is for the condition where the stroma of the urethra substitutes was reconstructed using only vesical fibroblasts (VF) and the standard protocol. Reseeding VF is for the condition where the stroma of the urethra substitute was reconstructed, seeding only VF, using the reseeding variation of self-assembly protocol (i.e. instead of stacking stromal sheets, fibroblasts were reseeded on the stromal sheet at mid-course of the production period, i.e. 14 days). The Hybrid VF is for the condition where the stroma of the substitute was reconstructed using only VF and a combination of the usual and reseeding protocols (3 sheets were reseeded on day 14 and stacked on day 28). (**A**) Thickness (in µm) was measured using ImageJ on images of Masson's trichrome-stained tissue sections. (**B**) Maximal strength of substitutes measured using Instron ElectroPuls E1000 (in N). The dotted line (0.27 N) represents the low limit value obtained with native human bladder tissue^26^. (**C**) Ultimate tensile strength calculated from data obtained using Instron ElectroPuls E1000 and thickness (in MPa). (**D**) Elastic modulus (inverse of elasticity) (in MPa). The dotted line (0.25 MPa) represents the superior limit value obtained with native tissue^26^. One-way ANOVA has been used to interpret the data. Asterisks indicate significant differences: (*) for p-value < 0.05, (**) for p-value < 0.01, (***) for p-value < 0.001, (****) for p-value < 0.0001, n = 4.

The first round of optimization has been performed using VF as a cell source. Three protocols to produce reconstructed tissues were compared: standard self-assembly^42^, reseeding^43^ and hybrid protocol^44^ combining the standard self-assembly and the reseeding techniques. All VF-derived tissues presented an inferior maximal strength and UTS but a superior elasticity compared to DF-based tissues (the elastic modulus is inversely proportional to the elasticity of tissues). Reseeding, especially within the hybrid protocol, allowed the production of more resistant tissues than the standard technique. However, the VF-based tissues remained unsuitable for clinical applications due to suboptimal values (Fig. 1). Therefore, the hybrid technique was used for all subsequent reconstructions due to its mechanical superiority.

### The addition of DF improved the mechanical strength of the flat urethral tissues

**Figure 2:**
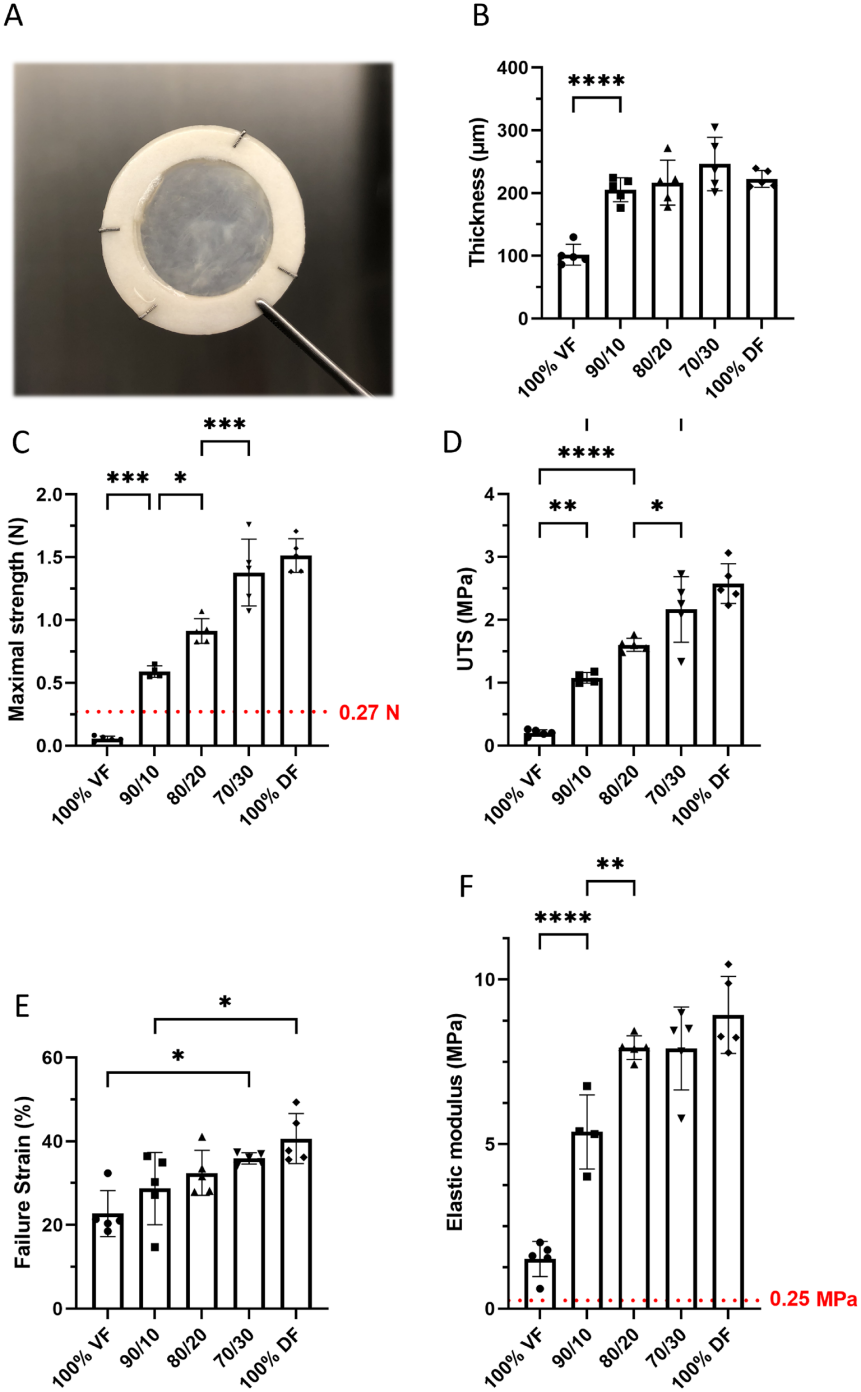
Characterization of the mechanical properties of the substitutes produced with different ratios of vesical or dermal fibroblasts. Each symbol represents the mean of measurements of one substitute. The error bars represent the mean +/− the standard deviation of the means. 100% VF is for the condition where the stroma of the urethra substitute was reconstructed using vesical fibroblasts (VF) only. The 90/10 is for a mix of 90% of VF and 10% dermal fibroblasts (DF), 80/20 is for a mix of 80% of VF and 20% DF, 70/30 is for a mix of 70% of VF and 30% DF, and 100% DF is for the condition where the stroma of the urethral substitute was reconstructed using only DF. All the substitutes were produced using the hybrid assembly technique. (**A**) Macroscopic image of a substitute produced in vitro. (**B**) Thickness of the substitutes (in µm) measured using ImageJ on images of Masson’s trichrome-stained tissue sections. (**C**) Maximal strength measured using Instron ElectroPuls E1000 (in N). The dotted line (0.27 N) represents the low limit value obtained with native tissue^41^. (**D**) Ultimate tensile strength (UTS) calculated from data obtained using Instron ElectroPuls E1000 and thickness (in MPa). (**E**) Failure strain in (%). (**F**) Elastic modulus (inverse of elasticity) (in MPa). The dotted line (0.25 MPa) represents the superior limit value obtained with native tissue^41^. Ordinary One-way ANOVA has been used to interpret the data. Asterisks indicate significant differences: (*) for p-value < 0.05, (**) for p-value < 0.01, (***) for p-value < 0.001, (****) for p-value < 0.0001, (n = 5).

To significantly enhance mechanical resistance, tissues were reconstructed using VF and DF at different ratios. Regardless of the cells used to reconstruct the stroma (DF or VF) or a mixture of both cells, all the substitutes had a similar macroscopic appearance (e.g., Fig. 2A). Tissues produced using VF/DF mixes showed increased tissue thickness compared to the condition where VF alone was used (Fig. 2B). Maximal strength, UTS and failure strain of the flat urethral tissues linearly increased (R^2^ ≥ 0.97) with decreasing VF/DF ratios (Fig. 2C–E). While all substitutes containing DF had maximal force values superior to those reported for native human bladder (dotted line)^41^, VF-only or 10% DF tissues had a higher elasticity than those produced with 100%, 30% or 20% DF (Fig. 2F).

### DF presence did not impact the pseudo-stratification of the urothelium but decreased the presence of polysaccharides

**Figure 3:**
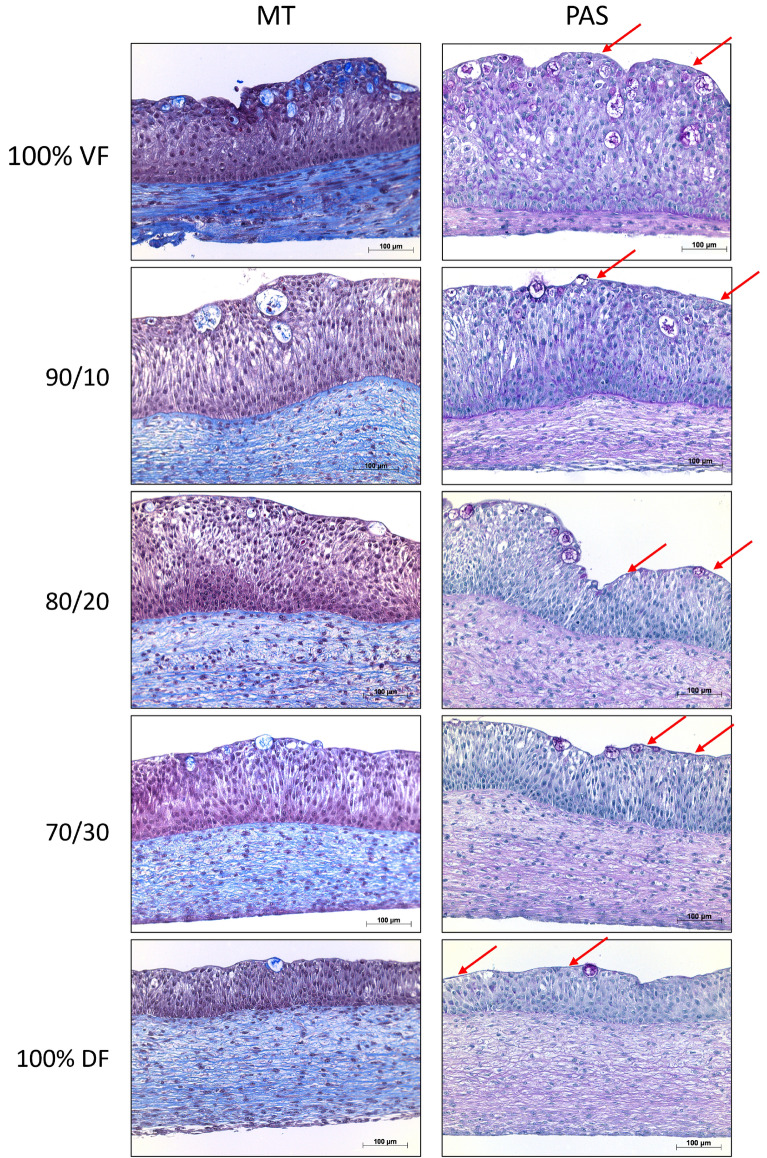
Histological appearance of the urethral substitutes. VF is for the condition where the stroma of the substitute was reconstructed using only vesical fibroblasts (VF). The 90/10 is for a mix of 90% of VF and 10% dermal fibroblasts (DF), 80/20 is for a mix of 80% of VF and 20% DF, 70/30 is for a mix of 70% of VF and 30% DF, and 100% DF is for the condition where the stroma of the urethra substitute was reconstructed using only DF. Representative photographs of Masson’s trichrome staining (MT, matrix in blue, cells in pink) or Periodic Acid Schiff stained (PAS, red arrows indicate the presence of polysaccharides at the apical region of the urothelium) tissue sections are presented, done in triplicate. The Masson’s trichrome staining allows to observe the collagen in blue, the cell cytoplasm in light red or pink, and the cell nuclei in dark brown to black. The PAS coloration stains the cell nuclei in blue and the polysaccharides, glycoproteins and glycolipids in magenta pink to red.

Varying the proportion of DF in the stroma of the urethral model during reconstruction did not significantly impact the histological features of the urothelium (Fig. 3, MT staining, purple). Indeed, all tissues showed a high level of urothelial organization with obvious pseudo-stratification (Fig. 3, left panel). It was also observed that the basal urothelial cells were better organized with a higher ratio of VF used for stroma production. PAS coloration highlighting the presence of polysaccharides showed a thick dark purple layer at the apical region of the urothelium (Fig. 3, right panel, red arrows). Additionally, mucous secreting cells could be observed in all substitutes, which contained polysaccharides (purple). Their presence seemed to correlate positively to the percentage of VF used for stroma production (Fig. 3, right panel).

### VF contribute to an improved barrier function of the flat urethral tissue

**Figure 4:**
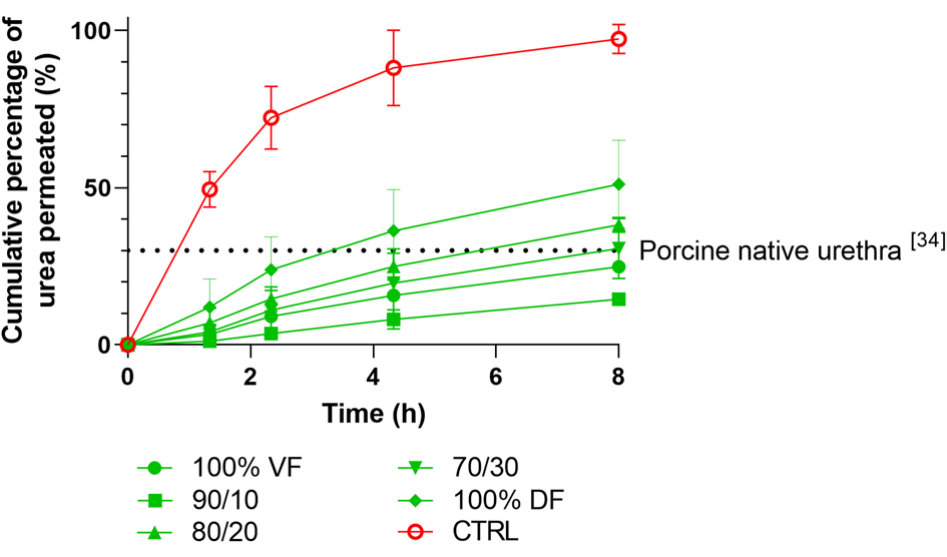
Barrier function of the urethral substitutes. Cumulated percentage of urea permeated (%) measured using a custom-made system. CTL is for the condition where the stroma of the urethra substitutes was reconstructed using only dermal fibroblasts (DF), and no UC was added. VF is for the condition where the stroma of the urethra substitute was reconstructed using only vesical fibroblasts (VF). The 90/10 is for a mix of 90% VF and 10% DF, 80/20 is for a mix of 80% VF and 20% DF, 70/30 is for a mix of 70% VF and 30% DF, and 100% DF is for the condition where the stroma of the urethra substitute was reconstructed using only DF, done in triplicate. The dotted line is the mean value obtained at eight hours for porcine native urethra^36^.

Barrier function was assessed through permeability assays of the substitutes, using stroma without urothelium as a negative control (Fig. 4, red curve). After eight hours, 100% of the urea poured into the donor chamber was found in the receiving chamber for this group (Fig. 4). However, the presence of UC significantly increased the impermeability of the flat urethral tissues, with a higher barrier function for the 90% and 100% VF tissues. Tissues made only with DF showed a reduced impermeability with 50% of the initial urea found in the receiving chamber at eight hours, whereas this quantity ranged from 10 to 20% for the 90% and 100% VF substitutes, respectively.

### Maturation of the uroplakin plaque is impacted by the composition of the stroma

**Figure 5:**
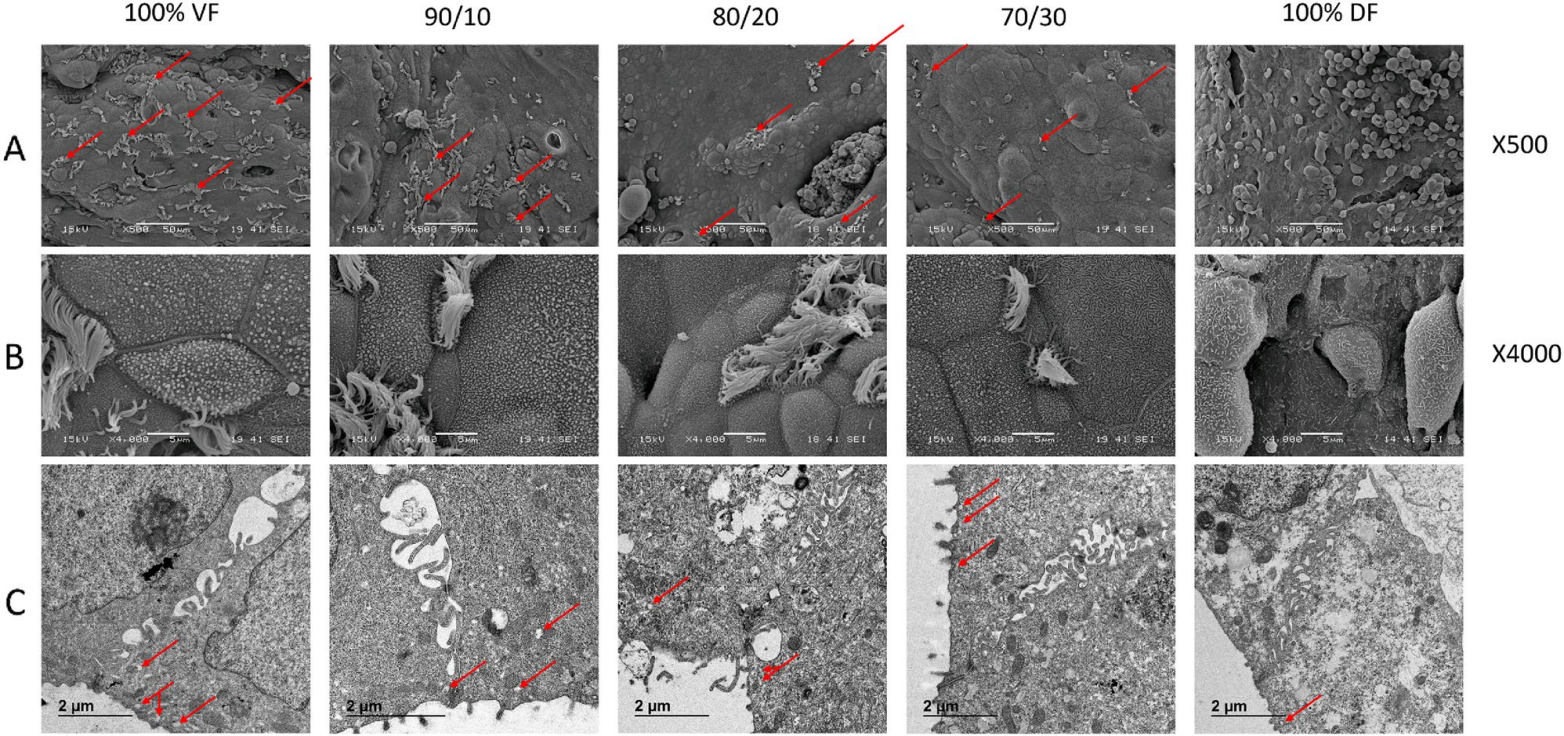
Ultrastructural characterization of urethral substitutes. (**A,B**) Urethra substitute samples were prepared and observed by scanning electron microscopy (SEM) and (**C**) transmission electron microscopy (TEM). VF is for the condition where the stroma of the urethral substitute was reconstructed using only vesical fibroblasts (VF). The 90/10 is for a mix of 90% of VF and 10% dermal fibroblasts (DF), 80/20 is for a mix of 80% of VF and 20% DF, 70/30 is for a mix of 70% of VF and 30% DF, and 100% DF is for the condition where the stroma of the urethral substitute was reconstructed using only DF, done in triplicate. Red arrows in (**A**) indicate microvilli observed in all substitutes. Red arrows in (**C**) indicate discoidal and fusiform vesicles observed in all substitutes.

Uroplakin plaque presence and maturation have been assessed using scanning and transmission electron microscopy. Superficial cells showed a polygonal outline with a flattened apical surface covered with short, thin microvilli of regular shape except for the DF-only substitute (Fig. 5A,B). Indeed, it was noticed that umbrella cells were absent in some locations for the DF substitutes, where intermediate cells were visible with a spherical shape. It can be noted that a higher proportion of DF correlated positively with a lower quantity of microvilli (Fig. 5A, red arrows). Discoid and fusiform vesicles, respectively, the less and most mature vesicles transporting uroplakins to the plasma membrane, were observed in all substitutes (Fig. 5C, red arrows). Tight junctions and desmosomes are also observable between umbrella cells.

### Among several urothelial-specific markers, only localization and intensity of K14 were modified by adding DF

**Figure 6:**
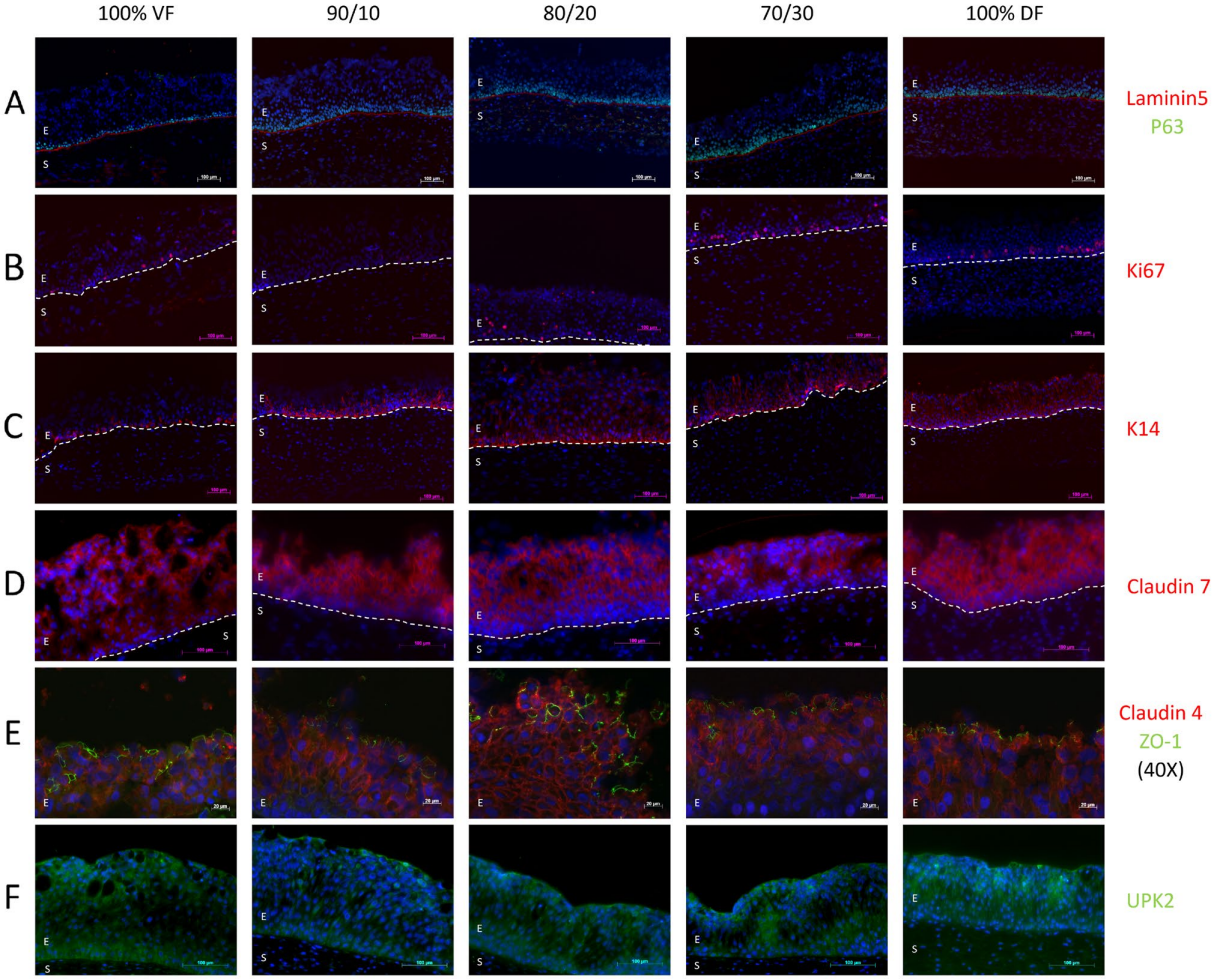
Molecular characterization of the substitutes. Samples were subjected to immunolabelling to detect specific proteins. VF is for the condition where the stroma of the urethra substitute was reconstructed using only vesical fibroblasts (VF). The 90/10 is for a mix of 90% of VF and 10% dermal fibroblasts (DF), 80/20 is for a mix of 80% of VF and 20% DF, 70/30 is for a mix of 70% of VF and 30% DF, and 100% DF is for the condition where the stroma of the urethra substitute was reconstructed using only DF, done in triplicate. E indicates the epithelial compartment of the substitute, and S, the stromal compartment. The dotted line represents the basal membrane between the stroma and the epithelium. Representative images were taken using a fluorescent microscope: (**A**) Co-labelling of laminin 5 (red) and p63 (green). (**B**) Labelling of Ki67. (**C**) Labelling of keratin 14 (K14). (**D**) Labelling of claudin 7. (**E**) Co-labelling of claudin 4 (red) and Zonula-Occludens 1 (ZO-1, green). (**F**) Labelling of uroplakin 2 (UPK2).

Adequate differentiation of the urothelium was evaluated following immunolabeling for specific markers selected to assess the localization and intensity of components from the basement membrane to the urothelium surface. The chosen markers were laminin 5 (laminin 332) (Fig. 6A, red) to stain the basement membrane and P63 (Fig. 6A, green), a transcription factor expressed by the urothelial progenitors in the basal layer of the urothelium, close to the basement membrane. Ki67 is present in the nucleus of proliferative cells (in G1, S, G2 and M phases) and expressed in the urothelium's basal layer (Fig. 6B). Keratin 14 (K14), a proliferative marker in the urothelium, was also present in the lower layers of the urothelium (Fig. 6C). Claudins, such as claudin-7, form tight junctions between epithelial cells. Claudin-7 presence was detected across all layers of the urothelium, emphasizing the intermediate and superficial layers (Fig. 6D). Claudin-4 (in red) was co-labelled with the ZO-1 protein (in green). The latter was present at the zonula occludens, likely contributing to tissue’s impermeability properties. While the distribution of claudin-4 was similar to claudin-7, the localization of ZO-1 was specific to the urothelium’s superficial layer (Fig. 6E,F). Finally, the presence of uroplakins was assessed with antibodies targeting UPK-2. Its presence has been detected globally in the urothelium, particularly in the superficial layer, where it appears as a thin line. Except for K14, all the markers showed a roughly similar localization and intensity for the substitutes reconstructed at different VF/DF ratios. The presence of K14 decreased with a lower DF percentage to resemble what is described for native urothelium for the tissues with a stroma produced with 100% VF (Fig. 6C).

## Discussion

It was previously shown that the use of DF to produce urologic substitutes provided tissues presenting great mechanical properties but also featuring the expression of some cutaneous markers^35^. On the other hand, when VF were used to produce substitutes, they presented an urothelium histologically similar to native tissue but presented mechanical properties too low to be clinically pertinent^39^. Therefore, protocols needed to be adapted. In this study, we determined the functional properties of substitutes produced using DF or VF alone or VF:DF mixes in ratios of 70%:30%, 80%:20% and 90%:10%.

For the current study, fibroblasts and epithelial cells were extracted from human bladder biopsies to produce the urethral substitute, representing a limitation. The ideal would have been to create transitional zones in the urothelium with the different morphologies found in the native urethra. However, in a clinical context, it is unfortunately almost impossible to safely harvest urethral cells from patients because it would induce significant comorbidities that could result in fibrosis and urethral stricture. Therefore, the main objective of this study was not to obtain urethral epithelium similar to the one found in native tissues but rather to obtain a substitute with the same functionality (and, therefore, impermeability). Thus, cells from the bladder have been chosen among all the epithelia available, even though they are not urethral cells. Indeed, bladder cells share many common characteristics with urethral cells. In our study, this epithelium allowed obtaining the optimal impermeability and, therefore, should best prevent the infiltration of urine in the underlying tissues. However, in future studies and as an alternative to bladder cells, it could be interesting to harvest urethral cells from patients undergoing gender confirmation surgery to establish accurate in vitro urethral models for fundamental research purposes.

To develop a strategy compatible with the clinical conditions, we wanted to evaluate a flat substitute rather than a tubular one. Indeed, in the clinic, flat biomaterials are the most commonly used (buccal mucosa, foreskin or skin flaps) to perform a urethroplasty. The flat graft is implanted on the penis of the patient (after skin incision) and then tubularized months after the initial implantation (2-step surgeries). Thus, the graft takes advantage of the underlying tissues to be vascularized and is well integrated with improved mechanical properties without the need to use a bioreactor during in vitro production.

Alternatively, if a flat substitute would be obtained by first producing a tubular model, the tube would then be cut open to lay it flat and allow the graft to take. Maturation in a bioreactor of a tubular self-assembled structure has already been performed and shown great results^36^. This strategy could have been used for the current study. However, using a bioreactor to mature the flat substitute would complicate the protocol, especially in a clinical context where the manufacturing protocol must be as simple as possible (aiming for low cost). Furthermore, the functionality and mechanical features of the biomaterial presented in this study are similar or higher (for the mechanical resistance) to the ones of native tissue, making using a bioreactor optional.

We observed that, independently of the self-assembly protocol variation used to produce the tissues, the use of VF-only did not allow to obtain mechanical properties sufficient to be comparable to those of native tissues. Then, we concluded that using 100% VF-derived substitutes in the clinic could not be possible due to the risk of tear during the surgeon's manipulation or suturing. On the other hand, DF-only substitutes produced with the standard self-assembly technique showed higher mechanical strengths but lower elasticity. This result led us to mix DF and VF to improve the mechanical characteristics of the reconstructed urethral substitutes.

Mechanical strength was proportionally enhanced by the addition of DF (0 to 30% DF; R^2^ = 0.98), but elasticity was reduced in a similar way (0 to 20% DF; R^2^ = 0.98) (Fig. 2). Therefore, the optimal choice for a graft should balance the elasticity required to resist without definitive deformation to the increase in pressure during micturition and the tissue’s strength. On this basis, the 90/10 and 80/20 mixes can be elected as suitable, as the maximal strength required of 0.27 N was achieved with higher elasticity than the other ratios. Indeed, with sufficient mechanical strength, it is expected that in vivo, the graft will be remodeled, and the surrounding tissues should increase their elasticity.

The histology of the tissues using MT staining showed a global aspect similar to native tissues (Fig. 3)^45,46^. The urothelium presented the characteristic pseudo-stratification of 5–8 layers, with basal, intermediate, and superficial cells, including an asymmetrical membrane at the surface of the urothelium (uroplakin plaque)^47^. According to the VF/DF ratio used, no apparent difference between the substitutes has been noted for the maturation degree of the urothelium. Nevertheless, round, hollow structures appear in the epithelium in increasing numbers as the percentage of VF used for the stroma production increases. These structures appear partly filled with material that may be secreted. PAS coloration highlighted the presence of polysaccharides enclosed in mucous-secreting cells, which were present in all the tested substitutes. These polysaccharides, like glycosaminoglycan (GAGs) or mucins, are known to cover the surface of the urothelium, mainly contributing to a defense mechanism role against microorganisms, toxic substances and carcinogens present in the urine^48^. They are then released at the apical membrane of the urothelium, where urine flow should help spread the different components. The presence of these mucous secreting cells seemed significantly reduced in the 70%:30% and 100% DF models, which could have impacted the functionality of the substitutes, and therefore explain the lower barrier function values obtained for these substitutes.

Most of the clinical failures of urological substitutes prepared by tissue engineering are due to poor differentiation of the urothelium, which cannot play its role as a barrier against urine, allowing it to infiltrate into the underlying tissues and inducing a fibrotic response. The barrier function of the substitutes was assessed using permeability tests using urea measurements. This molecule has been favored over others, such as labelled dextran beads used in several studies, because of its small size, which makes it possible to better demonstrate barrier tightness under closer physiological conditions. Results in this study were comparable to those obtained previously by our team using Franz cells to evaluate substitutes produced using the self-assembly protocol^36^. Indeed, in their study, whereas the permeability was complete for the stroma without epithelium (negative control), only 30% of the urea crossed the native porcine urethra after eight hours^36^. In this study, the only substitutes below this rate were the 100% VF and 90/10 ones, leading once again to the optimal choice toward the 90/10 mix.

Scanning electron microscopy provided an overview of the substitutes regarding maturation (Fig. 5). Orlandini et al. studied the male urethra using electron microscopy and described urothelial umbrella cells with polygonal outlines and a flattened apical surface with short microvilli^49^. In our study, the substitutes produced with 100% VF, 90/10, 80/20 and 70/30 mixes were very similar to those found in the literature for the native human urethra. In addition, the umbrella cells were visible on the surface of the tissue and were well-matured, providing the necessary impermeability. Still, a positive correlation may be observed (but not quantified) between the number of microvilli and the concentration of VF in the stroma, indicating a higher degree of epithelium maturation.

On the other hand, the presence of immature small spherical cells, and the absence of umbrella cells with smooth plaques on the superficial layer of the urothelium could be noted for the tissue produced with DF-only. These results correlated with the permeability results, explaining the lower functionality of the substitutes produced using only DF.

Transmission electron microscopy allowed us to observe the presence of discoid and fusiform vesicles in all substitutes, the latter being known to mediate the transport of uroplakins to cells apex^50^. These images also allowed observation of tight junctions between the umbrella cells, which participate in vivo to prevent urine from passing into the lower layers.

Molecular characterization of the substitutes was mainly achieved through immunofluorescence labelling against distinctive markers of the basement membrane and urothelium layers (Fig. 6). Laminin-5 (laminin 332) is a well-established marker of the basal lamina, the exchange zone separating the epithelium and stroma. The basal lamina is essential to maintain a strong adhesion of basal cells to the stroma and for the adequate development of the urothelium^51^. The basal cells were marked with the P63 antibody, a common basal layer progenitor cell marker^52^ and constitute a population of stem/progenitor cells with long-term proliferative activity^47,52^. Few cells of this basal layer also expressed Ki67, concurring with the hypothesis of proliferative potential. Stem cells are advantageous for grafts, as they will support long-term regenerative potential to the implanted tissues. In a healthy urothelium, K14 is present in the basal cell layer and absent in the upper layers^53^. This result could also be observed in the 100% VF, 90/10 and 80/20 mixes. For the lower VF/DF ratio, aberrant K14 expression in the upper layers was noted. A similar expression was found in a study on rat urothelium after injury but not in sham-treated rats^54^. These results show the importance of using organ-specific stromal cells in tissue engineering, as previously demonstrated^38,39^. This expression also points to the 90/10 and 80/20 mixes as the optimal compositions, as this percentage of DF does not seem to impact the K14 expression. As claudins are essential for maintaining tight junctions, claudin-7 and 4 have been evaluated in all substitutes. Claudins are found in the entire urothelium but are mainly expressed in the intermediate and superficial layers^55^. ZO-1 proteins are localized at the level of the tight junctions of the superficial layers^47^. Our results were consistent with the literature. The stroma composition did not seem to impact claudins and ZO-1 localization or expression patterns. Finally, UPK2, a hetero-tetramer forming UPK plaque^47^, was evaluated. UPKs positively correlate with cell maturation and are maximal in the terminally differentiated umbrella cells where UPKs form an asymmetric unit membrane^47^. In accordance with the literature, the presence of UPK2 was observed in all substitutes, particularly in the superficial layer. No difference was observed between the cell type ratios. The immunolabeling results confirmed that the substitutes produced with the 90%:10% and 80%:20% ratios represent substitutes with optimal features and functions.

Among the limitations of the current study, the use of bladder instead of urethral cells as already been cited, mainly due to the inherent risk of harvesting cells from the patient’s urethra. Furthermore, the substitute presented in the current study remains relatively more straightforward, as only two cell types have been used. Smooth muscle cells, pre-vascularization using endothelial cells or even a corpus spongiosum could be added to the substitute^37,46,56^. However, the substitute production protocol must be as simple as possible in a clinical context. Therefore, we limited the complexity to only two different cell types as it allows for obtaining mechanical resistance and functionality similar to the native tissues. We also must consider that a thicker tissue impedes graft take and leads to ischemia and secondary contraction.

## Conclusions

In conclusion, the substitutes produced with a ratio of 80 to 90% of VF mixed with DF showed good mechanical properties while featuring a near-native barrier function. Furthermore, no aberrant expression of urothelial markers was found in the substitutes, which may represent an excellent alternative for reconstructive surgery. The next step will be the in vivo implantation in a rabbit model of urethral replacement.

## Materials and methods

### Ethics statement

This study was conducted according to the Declaration of Helsinki and was approved by the institution’s committee for the protection of human participants (Comité d’éthique de la recherche du CHU de Québec-Université Laval, protocol number 2012-1341). All patients provided informed written consent prior to biopsies.

### Cell culture

Urothelial cells (UC) and VF were isolated from human bladder biopsies, whereas DF were isolated from human skin biopsies, as previously described^57–59^. Cells were stored frozen until thawed for utilization. They were used at passages 3 or 4 and cultured in Dulbecco-Vogt modification of Eagle’s medium (DMEM, Invitrogen, Burlington, Canada) containing 10% foetal bovine serum (Hyclone, Logan, UT), 100U/mL penicillin, and 25mg/mL gentamicin (Fb medium) (Fb medium). Epithelial cells were cultured in a medium containing a 3:1 mix of Dulbecco–Vogt modification of Eagle's (DMEM, Invitrogen, Burlington, Canada) and Ham’s F12 (Flow Lab., Mississauga, Canada) supplemented with 5% fetal bovine serum (FBS-H) (GE Healthcare, Chicago, IL), 24.3 μg/mL adenine (Corning), 5 μg/mL crystallized bovine insulin (Sigma Aldrich, St. Louis, MO, USA), 1.1 μM hydrocortisone (Teva Canada Ltd., Scarborough, Canada), 0.212 μg/mL isoproterenol hydrochloride (Sandoz Canada, Boucherville, Canada), 10 ng/mL epidermal growth factor (Austral Biologicals, San Ramon, CA, USA), and antibiotics; 100 U/mL penicillin and 25 mg/mL gentamicin (Sigma-Aldrich) (UC medium). Fibroblasts were cultured in DMEM supplemented with 10% fetal bovine serum (FBS) (Invitrogen) and antibiotics (Fb medium). All biopsies came from male donors (1 for each cell type).

### Production of 3D flat urethral tissues

The self-assembly technique was used to produce tridimensional reconstructed tissues, according to standard methods previously described^60^. Briefly, the fibroblasts were seeded to obtain a final concentration of 5.2 × 10^4^ cells/cm^2^ in 6-well plates (Falcon, ThermoFischer, USA) and cultivated in Fb medium supplemented with 50 µg/mL ascorbate. A second cell seeding was performed on day 14 on the newly formed cellularized stromal sheets for the reseeded or hybrid stroma models. The culture was pursued until enough neosynthesized ECM was assembled to form a manipulatable sheet, which commonly corresponded to 28 days. Next, three stromal sheets were superimposed for the standard self-assembly and hybrid tissues, and UC were seeded on top of the 3D tissues at a concentration of 5.2 × 10^4^ cells/cm^2^. Tissues were then cultured with UC medium supplemented with 50 µg/mL ascorbate. After seven days in submerged conditions, the flat urethral tissues were elevated at the air/liquid interface for 21 days to induce mature urothelium differentiation.

### Histological analyses

The flat urethral tissues were fixed with 3.7% formol and embedded in paraffin, sectioned (6 µm), and stained with Masson’s trichrome (MT) or Periodic Acid Schiff (PAS). Pictures were taken using a Zeiss Axio Imager M2 microscope equipped with an AxioCam ICc1 camera (Oberkochen, Germany).

### Immunolabeling

OCT-embedded cryosections  (5 μm thick) of flat urethral tissue were processed for immunofluorescence (IF). Antibodies were used at the indicated dilution (Table 1), and cell nuclei were stained with Hoechst solution (Sigma). At least ten tissue sections of each substitute (three per experimental group) were analyzed under a Nikon Eclipse E600 epifluorescence microscope (Nikon, Mississauga, Canada).

**Table 1:** Antibodies used for immunofluorescence.

Primary antibody	Company (Catalog #)	Concentration	Secondary antibody
Claudin-7	Thermofisher (349100)	1/100	Alexa-594 IgG anti-rabbit
Claudin-4	Thermofisher (329400)	1/200	Alexa-594 IgG anti-mouse
K14	Vector (VP-C410)	1/200	Alexa-594 IgG anti-mouse
Laminin 5 (laminin 332)	Chemicon (MAB19562)	1/400	Alexa-594 IgG anti-mouse
ZO-1	Thermofisher (40-2200)	1/200	Alexa-488 IgG anti-rabbit
P63	Abcam (Ab735)	1/200	Alexa-488 IgG anti-rabbit
Ki67	BD Pharmingen (556003)	1/200	Alexa-594 IgG anti-rabbit
UPK-2	MyBioSource (MBS460762)	1/100	Alexa-488 IgG anti-rabbit

### Mechanical testing

Mechanical properties of the flat urethral tissues were assessed by uniaxial tensile testing using an ElectroPuls E1000 mechanical tester (Instron Norwood, MA, USA). Bone-shaped biopsies were cut with a custom-made stainless-steel punch. Both extremities of the specimen were stretched at a constant rate of 0.2 mm/s until tissue ruptured. Strain curves were generated. Maximum strength (N) has been determined as the needed force to tear the substitute. The ultimate tensile strength (UTS) (MPa) was calculated by dividing the failure load by the section area of the tissues. The elastic modulus (MPa) was determined using the slope of the most linear region of the strain curve. The thickness of the stroma was assessed using an Axio Imager M2 microscope and analyzed by ImageJ software (NIH, Bethesda, MD). The entire tissue’s thickness was determined on the Masson’s trichrome stained sections using image J software (NIH, Bethesda, MD). One section per substitute and five substitutes per group were tested. All data were expressed as mean and standard deviation, and the graphs were generated using Microsoft Office Excel 2011 (Microsoft Corporation).

### Electron microscopy

Samples were processed as previously described^39^. Samples were fixed with 2.5% glutaraldehyde in 0.1 M cacodylate buffer (pH 7.4) at 4 °C, rinsed with cacodylate buffer and postfixed in 1% osmium tetroxide. The biopsies for transmission electron microscopy were stained with uranyl acetate, dehydrated through a graded series of ethanol, and embedded in Epon (Polysciences, Warrington, PA). They were cut in ultra-fine sections and counterstained with lead citrate and uranyl acetate. At least ten tissue sections of each construct were examined with a JEM 1230 (Tokyo, Japan). The biopsies for scanning electron microscopy were dehydrated, then critical point dried. At least ten tissue samples of each substitute were spattered with gold and viewed with a JEOL JSM-63060LV (Tokyo, Japan).

### Permeability test

Tissue permeability was evaluated using a custom-made technique similar to the Franz technique^61^. Briefly, the flat urethral tissue was placed on a 3 µm pore size 12-well plate insert with the urothelium facing the donor chamber. The exchange surface represents a surface of 0.2826 cm^2^. A glass cloning cylinder was pressed on the tissue to avoid any liquid leak, and a ring gasket was positioned around the cylinder to avoid movement. The insert was deposited on a 12-well plate. In the receiving chamber, 1000 μL of DMEM with 10% FBS was poured. Then, 150 µL of DMEM with 10% FBS containing 15 g/L of urea was poured into the cylinder on the top of the substitute (donor chamber). In 12-well plates, 1 mL of DMEM 10% FBS was added; therefore, the insert (receiving chamber) and the tissue were in contact with both media. The temperature of the chambers was maintained at 37 °C in 8% CO_2_. To evaluate the permeation rate, the bathing solution in the receiver compartment was removed and replaced by a fresh medium at selected time intervals (80, 140, 260 and 480 min). The urea concentration in the samples was determined using a clinical-grade urea-nitrogen detector (Dimension Vista®). The negative control (CTL) was the condition where the stroma of the substitutes was reconstructed using only DF, and no UC was added. The results were compared with the mean value obtained at eight hours for porcine native urethra^36^ (dotted line, Fig. 4) (n = 3).

### Statistics

Statistical analyses have been performed using the GraphPad Prism v.9.2 Software (San Diego, CA, USA). Results are expressed as mean and standard deviation. In addition, a one-way Analysis of Variance (ANOVA) was used to interpret the data.

## Data Availability

All data are available upon reasonable request. Please contact Dr Stéphane Bolduc: Stephane.Bolduc@fmed.ulaval.ca or Tel: 418-654-2282.

## References

[1] Keays MA, Dave S (2017). Current hypospadias management: Diagnosis, surgical management, and long-term patient-centred outcomes. Can. Urol. Assoc. J..

[2] Springer A, van den Heijkant M, Baumann S (2016). Worldwide prevalence of hypospadias. J. Pediatr. Urol..

[3] Nelson CP (2005). The increasing incidence of congenital penile anomalies in the United States. J. Urol..

[4] Baskin LS, Himes K, Colborn T (2001). Hypospadias and endocrine disruption: Is there a connection?. Environ. Health Perspect..

[5] Caldamone AA, Edstrom LE, Koyle MA, Rabinowitz R, Hulbert WC (1998). Buccal mucosal grafts for urethral reconstruction. Urology.

[6] Fu Q, Deng CL (2006). Ten-year experience with composite bladder mucosa-skin grafts in hypospadias repair. Urology.

[7] Bhargava S, Patterson JM, Inman RD, MacNeil S, Chapple CR (2008). Tissue-engineered buccal mucosa urethroplasty-clinical outcomes. Eur. Urol..

[8] Ehrlich RM, Alter G (1996). Split-thickness skin graft urethroplasty and tunica vaginalis flaps for failed hypospadias repairs. J. Urol..

[9] Song LJ, Xu YM, Hu XY, Zhang HZ (2008). Urethral substitution using autologous lingual mucosal grafts: An experimental study. BJU Int..

[10] Barbagli G, Selli C, Tosto A, Palminteri E (1996). Dorsal free graft urethroplasty. J. Urol..

[11] McAninch JW (2005). Urethral reconstruction: A continuing challenge. J. Urol..

[12] Dublin N, Stewart LH (2004). Oral complications after buccal mucosal graft harvest for urethroplasty. BJU Int..

[13] Djordjevic ML (2014). Graft surgery in extensive urethral stricture disease. Curr. Urol. Rep..

[14] Korneyev I, Ilyin D, Schultheiss D, Chapple C (2012). The first oral mucosal graft urethroplasty was carried out in the 19th century: The pioneering experience of Kirill Sapezhko (1857–1928). Eur. Urol..

[15] Nelson CP, Bloom DA, Kinast R, Wei JT, Park JM (2005). Long-term patient reported outcome and satisfaction after oral mucosa graft urethroplasty for hypospadias. J. Urol..

[16] Markiewicz MR, DeSantis JL, Margarone JE, Pogrel MA, Chuang SK (2008). Morbidity associated with oral mucosa harvest for urological reconstruction: An overview. J. Oral Maxillofac. Surg..

[17] Aldaqadossi HA, Shaker H, Youssof H, Kotb Y, Eladawy M (2019). Outcomes of staged lingual mucosal graft urethroplasty for redo hypospadias repair. J. Pediatr. Urol..

[18] Spilotros M (2017). Buccal mucosal graft urethroplasty in men-risk factors for recurrence and complications: A third referral centre experience in anterior urethroplasty using buccal mucosal graft. Transl. Androl. Urol..

[19] Nelson CP, Bloom DA, Kinast R, Wei JT, Park JM (2005). Patient-reported sexual function after oral mucosa graft urethroplasty for hypospadias. Urology.

[20] Hmida W, Othmen MB, Bako A, Jaidane M, Mosbah F (2019). Penile skin flap: A versatile substitute for anterior urethral stricture. Int. Braz. J. Urol..

[21] Caneparo CS, Bolduc S (2019). Challenges and perspectives in male anterior urethra reconstruction using tissue engineering. Urol. Res. Therap. J..

[22] Atala A (2017). The potential role of tissue-engineered urethral substitution: Clinical and preclinical studies. J. Tissue Eng. Regen. Med..

[23] Engel O (2012). 15 Tissue—Engineered buccal mucosa urethroplasty. Outcome of our first 10 patients. J. Urol..

[24] Barbagli G (2018). Anterior urethroplasty using a new tissue engineered oral mucosa graft: Surgical techniques and outcomes. J. Urol..

[25] Fossum M, Skikuniene J, Orrego A, Nordenskjold A (2012). Prepubertal follow-up after hypospadias repair with autologous in vitro cultured urothelial cells. Acta Paediatr..

[26] Osman NI, Patterson JM, MacNeil S, Chapple CR (2014). Long-term follow-up after tissue-engineered buccal mucosa urethroplasty. Eur. Urol..

[27] Abbas TO, Yalcin HC, Pennisi CP (2019). From acellular matrices to smart polymers: Degradable scaffolds that are transforming the shape of urethral tissue engineering. Int. J. Mol. Sci..

[28] Raya-Rivera A (2011). Tissue-engineered autologous urethras for patients who need reconstruction: An observational study. Lancet.

[29] Ram-Liebig G (2017). Results of use of tissue-engineered autologous oral mucosa graft for urethral reconstruction: A multicenter, prospective, observational trial. EBioMedicine.

[30] Atala A, Guzman L, Retik AB (1999). A novel inert collagen matrix for hypospadias repair. J. Urol..

[31] El-Kassaby AW, Retik AB, Yoo JJ, Atala A (2003). Urethral stricture repair with an off-the-shelf collagen matrix. J. Urol..

[32] Fiala R, Vidlar A, Vrtal R, Belej K, Student V (2007). Porcine small intestinal submucosa graft for repair of anterior urethral strictures. Eur. Urol..

[33] El-Kassaby A, AbouShwareb T, Atala A (2008). Randomized comparative study between buccal mucosal and acellular bladder matrix grafts in complex anterior urethral strictures. J. Urol..

[34] Saba I, Jakubowska W, Bolduc S, Chabaud S (2018). Engineering tissues without the use of a synthetic scaffold: A twenty-year history of the self-assembly method. Biomed. Res. Int..

[35] Magnan M (2009). Tissue engineering of a genitourinary tubular tissue graft resistant to suturing and high internal pressures. Tissue Eng. A.

[36] Cattan V (2011). Mechanical stimuli-induced urothelial differentiation in a human tissue-engineered tubular genitourinary graft. Eur. Urol..

[37] Imbeault A (2013). An endothelialized urothelial cell-seeded tubular graft for urethral replacement. Can. Urol. Assoc. J..

[38] Carrier P (2009). Impact of cell source on human cornea reconstructed by tissue engineering. Investig. Ophthalmol. Vis. Sci..

[39] Bouhout S, Chabaud S, Bolduc S (2016). Organ-specific matrix self-assembled by mesenchymal cells improves the normal urothelial differentiation in vitro. World J. Urol..

[40] Masri C, Chagnon G, Favier D, Sartelet H, Girard E (2018). Experimental characterization and constitutive modeling of the biomechanical behavior of male human urethral tissues validated by histological observations. Biomech. Model Mechanobiol..

[41] Dahms SE, Piechota HJ, Dahiya R, Lue TF, Tanagho EA (1998). Composition and biomechanical properties of the bladder acellular matrix graft: Comparative analysis in rat, pig and human. Br. J. Urol..

[42] Auger, F. A., Remy-Zolghadri, M., Grenier, G. & Germain, L. A truly new approach for tissue engineering: The LOEX self-assembly technique. In *Ernst Schering Res Found Workshop*, 73–88. 10.1007/978-3-662-04816-0_6 (2002). 10.1007/978-3-662-04816-0_6 11816275

[43] Chabaud S, Rousseau A, Marcoux TL, Bolduc S (2017). Inexpensive production of near-native engineered stromas. J. Tissue Eng. Regen. Med..

[44] Jakubowska W (2020). Prevascularized tissue-engineered human vaginal mucosa: In vitro optimization and in vivo validation. Tissue Eng. A.

[45] Natali AN (2017). Mechanics of the urethral duct: Tissue constitutive formulation and structural modeling for the investigation of lumen occlusion. Biomech. Model Mechanobiol..

[46] Mikami H (2012). Two-layer tissue engineered urethra using oral epithelial and muscle derived cells. J. Urol..

[47] Dalghi MG, Montalbetti N, Carattino MD, Apodaca G (2020). The urothelium: Life in a liquid environment. Physiol. Rev..

[48] Parsons CL, Boychuk D, Jones S, Hurst R, Callahan H (1990). Bladder surface glycosaminoglycans: An epithelial permeability barrier. J. Urol..

[49] Orlandini SZ, Orlandini GE (1989). Ultrastructure of human male urethra. Arch. Androl..

[50] Hudoklin S, Jezernik K, Neumuller J, Pavelka M, Romih R (2012). Electron tomography of fusiform vesicles and their organization in urothelial cells. PLoS ONE.

[51] Rousseau A (2015). Adipose-derived stromal cells for the reconstruction of a human vesical equivalent. J. Tissue Eng. Regen. Med..

[52] Wang C, Ross WT, Mysorekar IU (2017). Urothelial generation and regeneration in development, injury, and cancer. Dev. Dyn..

[53] Papafotiou G (2016). KRT14 marks a subpopulation of bladder basal cells with pivotal role in regeneration and tumorigenesis. Nat. Commun..

[54] Kullmann FA (2017). Urothelial proliferation and regeneration after spinal cord injury. Am. J. Physiol. Renal Physiol..

[55] Acharya P (2004). Distribution of the tight junction proteins ZO-1, occludin, and claudin-4, -8, and -12 in bladder epithelium. Am. J. Physiol. Renal Physiol..

[56] de Graaf P (2018). The multilayered structure of the human corpus spongiosum. Histol. Histopathol..

[57] Ringuette Goulet C (2018). Exosomes induce fibroblast differentiation into cancer-associated fibroblasts through TGFbeta signaling. Mol. Cancer Res..

[58] Ringuette Goulet C (2017). Tissue-engineered human 3D model of bladder cancer for invasion study and drug discovery. Biomaterials.

[59] Germain L (1993). Improvement of human keratinocyte isolation and culture using thermolysin. Burns.

[60] Bouhout S (2010). In vitro reconstruction of an autologous, watertight, and resistant vesical equivalent. Tissue Eng. A.

[61] Supe S, Takudage P (2021). Methods for evaluating penetration of drug into the skin: A review. Skin Res. Technol..

